# Evaluating the Phytoremediation Potential of *Eichhornia crassipes* for the Removal of Cr and Li from Synthetic Polluted Water

**DOI:** 10.3390/ijerph20043512

**Published:** 2023-02-16

**Authors:** Muhammad Umar Hayyat, Rab Nawaz, Ali Irfan, Sami A. Al-Hussain, Mehlil Aziz, Zafar Siddiq, Sajjad Ahmad, Magdi E. A. Zaki

**Affiliations:** 1 Sustainable Development Study Centre, Government College University, Lahore 54000, Pakistan; 2 Department of Environmental Sciences, The University of Lahore, Lahore 54000, Pakistan; 3 Department of Chemistry, Government College University Faisalabad, Faisalabad 38000, Pakistan; 4 Department of Chemistry, College of Science, Imam Mohammad Ibn Saud Islamic University (IMSIU), Riyadh 13623, Saudi Arabia; 5 Department of Botany, Government College University, Lahore 54000, Pakistan; 6 Civil Engineering Department, Sahiwal Campus, COMSATS University Islamabad, Sahiwal 57000, Pakistan

**Keywords:** water management, pollutant removal, water pollution and remediation, emerging pollutant, heavy metals

## Abstract

Heavy metals like chromium (Cr) are hazardous pollutants for aquatic life in water bodies. Similarly, lithium (Li) is also an emerging contaminant in soil and water which later is taken up by plants. The aim of the present study is to evaluate the removal rate of Cr and Li by *Eichhornia crassipes*. The rate of the removal of Cr and Li by roots, stems, and leaves of *E. crassipes* were evaluated. The translocation factor (TF) and bioaccumulation factor (BAF) were also estimated. Roots of *E. crassipes* accumulated higher concentrations of Cr and Li as compared to the stems and leaves. BAF for Cr and Li showed that *E. crassipes* effectively accumulated the Cr and Li in the roots as compared to the stems and leaves. Statistical analysis showed that *E. crassipes* removed significant concentrations of Cr and Li (*p* ≤ 0.05). Thus, this study recommends that Cr and Li can be effectively removed by *E. crassipes*. High concentrations of Cr and Li could also be removed by *E. crassipes*. This technology could be used for the cleanup of the environment because it is eco-friendly and cost-effective.

## 1. Introduction

The industrial revolution has increased the threat of heavy metals and other contaminants in the environment. These metals not only enter the food chain but also cause serious threats to human health [1]. Environmental pollution has also been increasing in the last few years due to urbanization, industrialization, and an increase in the concentration of heavy metals in different environmental media, which are causing major threats all over the world [2]. Many commercial industries discharge wastewater without any treatment into the water bodies. This results in the release of huge amounts of lead, chromium, nickel, cadmium, mercury, arsenic, uranium, copper etc., into the receiving environment. Some metals are required in small quantities for the biological metabolism of living organisms, and these metals become toxic to flora and fauna by entering into the food chain [3]. Many physical, chemical and biological processes have been discovered up till now. Many techniques are also used to remove water pollution, which also mitigates the issues of water shortages through the reuse of water. Toxic metals are removed by the use of physical methods like crushing, drying, filtration, sedimentation, metal ion absorption and sieving. These physical methods are cheap, but their efficiency (50%) is questionable [4].

An effective plant-based approach that is called phytoremediation was carried out by using plants to remove and extract pollutants from the environment [5]. There are many advantages to using this approach; (1) it is an autotrophic system that uses solar energy so that it can be simply managed, and its maintenance and installation costs are very low, so it is economically feasible. (2) the pollution in the ecosystem and environment can be reduced, so it is eco-friendly; (3) phytoremediation can be applied on a large scale and can be disposed of easily, (4) the stability of heavy metals is increased by reducing metal leaching, hence reducing the spread of contaminants [6]. Various species of aquatic plants belong to different families like Hydrocharitaceae, Lemnaceae, Potamogetonaceae, Ranunculaceae, Typhaceae, Cyperaceae, Najadaceae, Zosterophyllaceae, Haloragaceae, and Pontederiaceae. These are used in the phytoremediation of wastewater [7]. The pollutants of organic and inorganic nature from water are accumulated in different plant species by using field and hydroponic applications [7].

So the major advantages of phytoremediation over other bioremediation and biomaterial-based waste treatment methods are; low cost, based on natural and renewable resources, production of feedstock for various uses, insignificant carbon footprint and effectiveness in a wide range of environmental conditions [8]. The use of bacteria and fungi has a number of limitations in their wider role in bioremediation as compared to phytoremediation. Microbes are highly sensitive to pH and temperature. Biological factors like interaction and composition of the community are key to completing the process of bioremediation. The operational cost of such methods is high [9,10]. Environmental factors and the separation of algae biomass from the treated water are major constraints in the use of algae for the treatment of wastewater [11]. Physical, chemical or both modifications are needed to use none living biomass for the removal of pollutants. Another limitation is the cost either for modifications or already commercial use of the considered none living biomass [12,13].

Contaminants, including heavy metals, are effectively removed from water by a variety of plant species, and this process is known as phytoremediation. This type of remediation has gained much importance among government bodies, non-government bodies, and scientists. The use of plants for the remediation of wastewater started 300 years ago [14]. *Eichhornia crassipes*, commonly known as water hyacinth, belong to the Pontedriaceae. It is considered a free-floating and highly productive aquatic plant that originated in South America. It is considered the most toxic weed due to its high rate of growth, great resistance to pollution, and enormous capacity for the absorption of nutrients [15]. There are many important uses for water hyacinths, but one of the most important uses is wastewater treatment. It has a great capacity for the accumulation of different heavy metals [16,17]. Chromium is considered an essential element for the growth and development of humans, but chromium, having hexavalent valency, is the most toxic element found in the earth’s crust [18]. The drinking water standard for total chromium is 0.1 mg/L or ppb according to USEPA (EPA 816-F-09-004). The level of Cr in drinking water should be 0.05 mg/L [19]. Chromium metal is mostly present in the wastewater discharged from agricultural, industrial, transportation and mining activities. It is highly fatal to all natural ecosystems. Tanning processes involve high concentrations of chromium salts, but a large quantity of chromium is converted into sludge [20]. Anthropogenic and natural activities lead to the transport of chromium into ground and surface water [21]. The pollution of hexavalent chromium is mostly related to sludge and wastewater from different industries like metal finishing, textile processing and dyeing, tanning, and the leather industry [22]. The exposure of lithium to animals and humans has been increasing day by day because of improper use and disposal of products that contain lithium. The production of lithium is 77,000 tonnes per year globally [23], and this has increased three times since 2000 [24]. The demand for lithium has increased due to electric vehicles and electronic products [25]. Water and soil are contaminated by lithium present in drugs and ceramics from stormwater, landfill leachates, and sewage [26]. Human health is critically at risk due to the high concentration of Li in soil and water in different regions of the world [27]. High concentrations of lithium (more than 1.4 mmol Li/L in body serum) can damage renal, cardiovascular and neurological systems, so this is the reason that Li must be controlled in fresh water and natural environment. Besides this, there is no limit set by the EPA for the maximum concentration of Li in drinking water [28]. WHO has also not set any limit on Li in the drinking water. Very few reports are present that mention lithium in the aquatic and terrestrial environment. The large-scale use of lithium in the energy sector has increased the amount of Li contamination in the environment.

The main objectives of the present study were to define the potential of *Eichhornia crassipes* for the removal of chromium (Cr) and lithium (Li), to determine the differences in accumulation of chromium and lithium by stems, leaves and roots of this macrophyte, to compare the removal rate of chromium and lithium by *Eichhornia crassipes* and to encourage the methods like phytoremediation to treat contaminated water because they are cost-efficient, easy to control and very effective. In this study, water hyacinth was used to evaluate the removal rate of lithium and chromium. In recent years, Li has become an emerging pollutant on a global scale. The importance of Li is increasing rapidly on a worldwide scale because of its growing and excessive use. It has various sources in nature, such as ores, brines, smelting and mining; however, the source in context with the present study is an artificial one, i.e., Li-ion batteries.

Anthropogenic and natural activities lead to the transport of chromium into ground and surface water [22]. Chromium is also released into the environment by some other industries like leather, metal cleaning, pharmaceuticals, paint, cement, and galvanization in different chemical and physical states that result in the accumulation of metals in plants, fishes, clams, crabs, etc. [29]. There is no work available on Li impacts on the growth and physiological parameters of *Eicchornia crassipes* in Pakistan. Internationally as well, there are only a few studies on this subject matter. The removal rate of Cr by *Eichhornia* is also determined because Cr is released by many industries and is causing a lot of water pollution. So, Cr is causing many health issues in humans [30]. *Eichhornia crassipes* is also the main element of interest because of its high growth rate. This plant species is causing many problems for irrigation, fishing and navigation in the coastal areas, but studies are conducted to use it for environmental and economic benefits. This study focuses on the removal of chromium and lithium from the aquatic environment. A comparison between chromium and lithium was also made to determine which elements can be effectively removed by water hyacinth. The removal rate of lithium by *Eichhornia crassipes* is not reported in the literature, although it is an emerging pollutant and causes many problems in the environment. Therefore, this study emphasizes examining this aquatic plant with respect to metal contamination and one of the emerging pollutants (Li).

## 2. Materials and Methods

### 2.1. Preparation of Micro and Macronutrients

The concentrations used for the experiment in the case of Cr were 2, 4, 6, and 8 mg/L. For Li and were 10, 20, 30 and 40 mg/L and the combination of Cr and Li was also used in 2, 4, 6 and 8 mg/L concentrations. Then, the solutions of micro and macronutrients are prepared. The solution of micro nutrients were KCl, H_3_BO_3_, MnSO_4_ · H_2_O, ZnSO_4_.7H_2_O, CuSO_4_.5H_2_O, H_2_MoO_4_ (85% MoO_3_), NaFe EDTA (10%Fe) and the solution of macronutrients were KNO_3_, Ca(NO_3_).4H_2_O, NH_4_H_2_PO_4_ and MgSO_4_.7H_2_O. From these micro and macronutrients, Hoagland’s solution was prepared [31].

### 2.2. Preparation of Cr and Li Doses

First of all, the salt of potassium dichromate (K_2_Cr_2_O_7_) was weighed according to concentrations of 2, 4, 6 and ppm of Cr; similarly, lithium Chloride (LiCl) was weighed according to the concentrations of 10, 20, 30, and 40 ppm of Li. The concentrations of Cr and Li were weighed and preserved in 8 airtight zipper bags (4 for Cr and 4 for Li). For 2, 4, 6 and 8 ppm of Cr + Li, salts of Cr and Li were also weighted. In this way, a total of 36 packets of LiCl salt was weighed and stored at room temperature. For weighing the salt, Electric Balance (AUY220) was used.

### 2.3. Macrophytes Collection

On the basis of phytomorophological properties, macrophytes were collected from the botanical garden. Healthy and fresh plants were collected and kept in a water bath. The plants were then brought to the laboratory and, after washing them with tap water, kept under suitable environmental conditions.

### 2.4. Experimental Set Up

A total of 39 containers having a capacity of 5 L were selected and filled with 4 L of water. Plants having equal size and weight were selected. One plant was added to each container for 15 days in the winter, spring and summer seasons. Three containers were used as a control for having nutrients and macrophytes only. 20 mL of Hoagland’s solution was added to each container. Metal concentrations were added to all the containers other than the control. A triplet of each concentration was used for accurate results. Distilled water was added to compromise the effect of evapotranspiration during the whole experiment.

### 2.5. Height and Weight of Plants

Before applying the doses of salt, the height of each replicate was measured in centimetres using a measuring tape. This was done to record data on the condition of the plants before applying doses of Cr and Li so that it could be compared with the data afterwards. Hence the impacts of Cr and Li in these plants could be documented. Similarly, the fresh weight in grams of each plant was measured before the application of salt and after 15 days of each setup when salt was applied.

### 2.6. Chlorophyll Content and Physiological Parameters

After measuring the height and weight of the plant, the chlorophyll content of the leaves of each replicate was also determined and recorded using a chlorophyll meter (502 SPAD Spectrum). After that, by using an Infrared Gas Analyzer (IRGA) of the model LCA4 (ADC, Reading, UK), the rate of transpiration, rate of photosynthesis, and stomatal conductance of leaves of each replicate was determined, among other readings of gas exchange parameters. The readings were taken in ambient conditions in the morning time between 9:00 a.m. to 11:00 a.m. after 15 days of each setup.

The aforementioned readings of chlorophyll content and other physiological parameters were recorded after applying the salinity treatment. Moreover, both the chlorophyll meter and IRGA are in good working condition and are currently present in Botany Department, GCU, Lahore.

### 2.7. Harvesting of Plants

All the plants under both Cr and Li stresses were harvested from the roof of SDSC with proper care. They were harvested along with their intact roots, wrapped with wet tissue paper, packed in plastic bags, were labelled accordingly. After that, the plants were taken to the laboratory for further analysis.

### 2.8. Determination of Cr and Li Concentration

Oven-dried roots, shoots, and leaves of each treatment of Cr and Li were weighed 5 g each and shifted in crucibles. The crucibles were labelled according to each treatment and parts of *E. crassipes*. Then, they were placed in a muffle furnace (Carbolite) CWF1200 at 450 °C for 2 h. After they were cooled down, the crucibles were taken out, and the ash of each treatment was dissolved in 20 mL of distilled water. The distilled water had already been dissolved in 0.01N HNO_3_ [32,33]. After dissolving the ash, the solutions were filtered using filter papers and stored in plastic bottles. Then, the concentration of Cr and Li in root stems and leaves for each treatment of *E. crassipes* was determined using Automatic Flame Photometer (Model S20, ADC, Reading, UK) present in good working condition in the Department of SDSC in GCU, Lahore.

For Cr and Li, the translocation factor from roots to the leaves will be collected by:

TF = Cl/Cr (1)

The value of a translocation factor greater than 1 means that there is an effective translocation of metal from roots to the leaves. To determine the bioaccumulation factor, the following formula will be used:

BAF = Cp/Cm (2)

Here, Cp is the metal concentration in a plant, while Cm is the metal concentration in the medium. The value of a bioaccumulation factor greater than 1 means that the macrophyte is a bio-accumulator. On the other hand, a value greater than 10 shows that macrophyte is classified as a hyperaccumulator [32,33].

### 2.9. Statistical Analysis of Data

After the preparation of samples, the metal analysis was conducted, and the data was put in one-way ANOVA by the use of SPSS; various comparisons were made, in that *p* ≤ 0.05 was taken as an important value for the experimental analysis. In addition, mean values (±SE) of all the treatments were compared one by one with the control.

## 3. Results

### 3.1. Removal of Cr and Li

**Figure 1:**
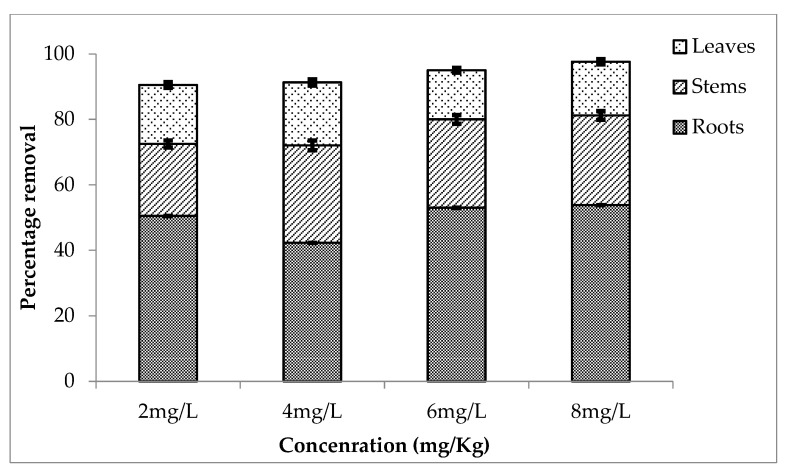
Mean (± SE) percentage removal of chromium by roots, stems and leaves of *Eichhornia crassipes* during first setup where *p* ≤ 0.05.

The removal of chromium (Cr) and lithium (Li) by *Eichhornia crassipes* and shown below. The removal rate of Cr and Li by the roots, stems and leaves of *E. crassipes* with their statistical analysis is shown. The concentrations of chromium removed by *Eichhornia crassipes* during first (20 January 2022–5 February 2022), second (15–30 March 2022) and third (30 May 2022–15 June 2022) setup are shown below. It is clear from the results that there is maximum absorption of heavy metal by the roots as compared to the stem and leaves. It is shown that the control plant had no concentration of metal in it while plants having treatments of 2, 4, 6 and 8 mg/L showed maximum uptake of metal from water to different parts of the plant. These concentrations are effectively removed by *E. crassipes.* The percentage removal of chromium during first setup by roots stems and roots are shown in Figure 1. It is shown that there is maximum absorption of chromium at high concentration (8 mg/L) as the removal rate is 53.8% by roots, 27.4% by stems and 16.4% by the leaves.

**Figure 2:**
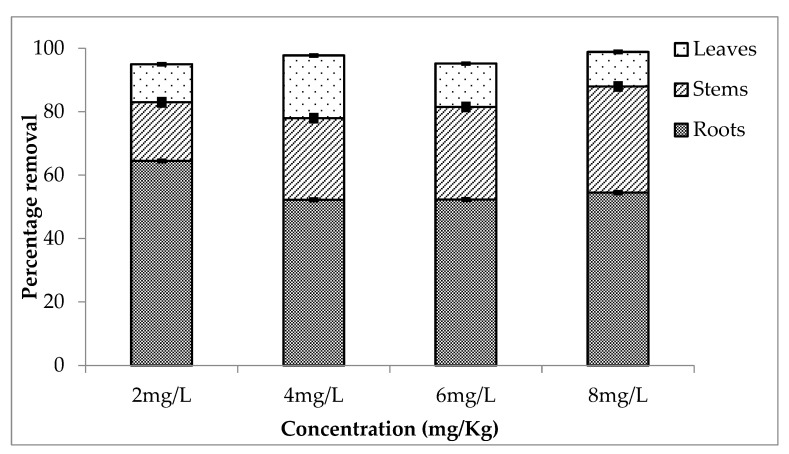
Mean (± SE) percentage removal of chromium by roots, stems and leaves of *Eichhornia crassipes* during second setup where *p* ≤ 0.05.

During second setup, there is a relatively high rate of removal as compared to the first setup. The percentage removal of chromium during second setup is shown in Figure 2. Roots accumulated high concentration; i.e., 65% chromium was absorbed by root for 2 mg/L treatment, and 53, 53 and 55% were removed by roots in case of 4, 6 and 8 mg/L treatments. The rate of removal was high from the water having 8 mg/L concentration (55, 34 and 11% by roots, stems and leaves, respectively).

**Figure 3:**
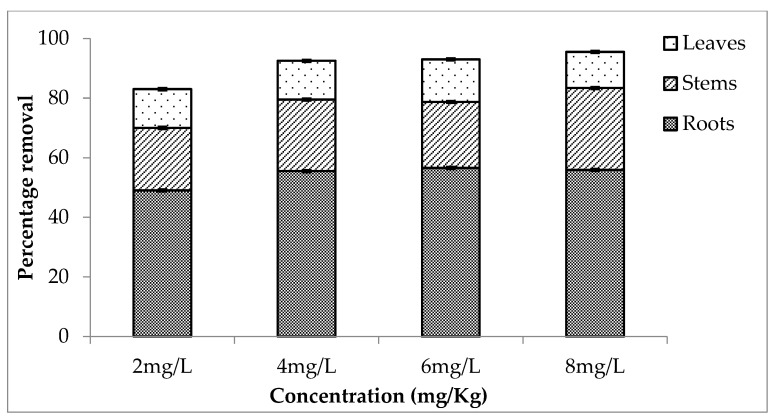
Mean (± SE) percentage removal of chromium by roots, stems and leaves of *Eichhornia crassipes* during third setup where *p* ≤ 0.05.

The rate of removal was relatively low in the third setup, which was held from 30 May to 15 June 2022, as compared to the first and second setups. Figure 3 shows the percentage removal proving that roots accumulated ore concentration of metal as compared to the aerial parts of the plant. The rate of removal by the roots was 49, 56, 57 and 56% from 2, 4, 6 and 8 mg/L. It is clear from the comparison that the rate of removal is high in the case of second setup. The plant showed high efficiency of 96, 98, 95 and 99% for 2, 4, 6 and 8 mg/L concentrations.

**Figure 4:**
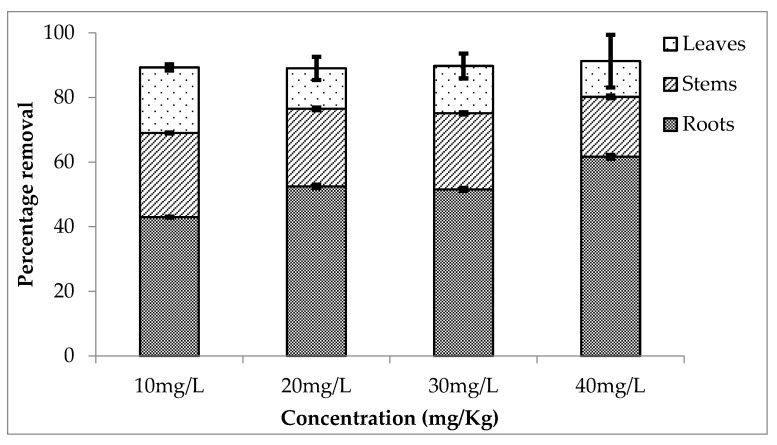
Mean (± SE) percentage removal of lithium by roots, stems and leaves of *Eichhornia crassipes* during first setup where *p* ≤ 0.05.

The rate of removal of one of the most widely used metalloid lithium is observed by the use of *Eichhornia crassipes*. It is highly efficient for the removal of lithium. The rate of removal was also observed during three different setups around the year. Figure 4 shows the percentage removal of lithium by different parts of the plant. Roots accumulated more concentration of lithium as compared to the stems and leaves. It is observed that 43, 53, 52 and 62% lithium was removed from 10, 20, 30 and 40 mg/L concentrations. At the same time, stem and leaves absorbed 26, 24, 24, 19% and 20, 13, 15, and 11%, respectively. An almost equal amount of lithium is removed by the plant from each treatment.

**Figure 5:**
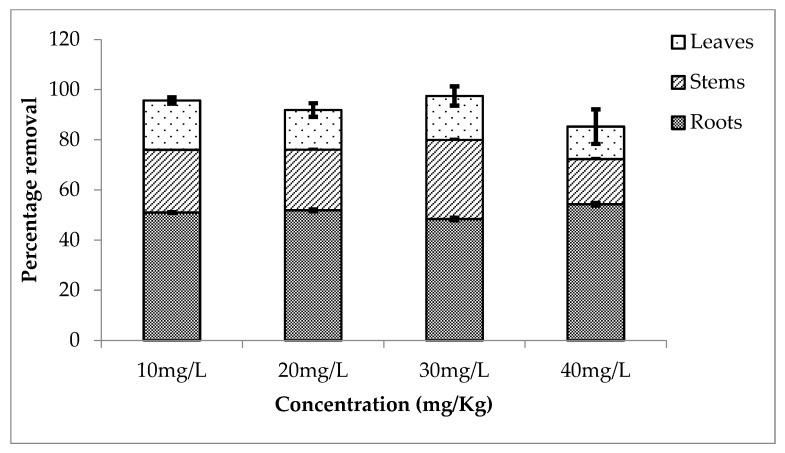
Mean (± SE) percentage removal of lithium by roots, stems and leaves of *Eichhornia crassipes* during second setup where *p* ≤ 0.05.

The second setup showed more percentage removal of lithium as compared to the other two setups. Figure 5 shows the percentage removal by roots, stems and leaves. Roots absorbed 51, 52, 48 and 54% from 10, 20, 30 and 40 mg/L. While 25, 24, 31, 18% and 20, 16, 18, and 13% lithium was absorbed by stems and leaves, respectively. Overall this setup showed a removal rate of about 96, 92, 97 and 95% (for 10, 20, 30 and 40 mg/L, respectively).

**Figure 6:**
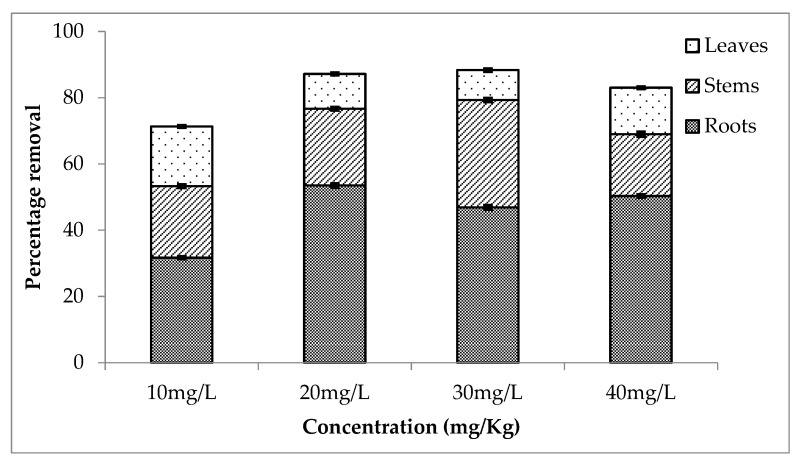
Mean (± SE) percentage removal of lithium by roots, stems and leaves of *Eichhornia crassipes* during third setup where *p* ≤ 0.05.

Figure 6 shows the average removal of lithium and percentage removal during the third setup. The percentage removal was 32, 54, 47 and 50% (by roots), 22, 23, 32 and 19% (by stems) and 18, 11, 9 and 14% (by leaves) for 10, 20, 30 and 40 mg/L. The rate of removal is relatively high compared to the other two setups.

Overall comparison of lithium removal showed that lithium is effectively removed during the second setup. For 10, 20, 30 and 40 mg/L, the percentage removal during second setup was 96, 92, 96 and 85% respectively, which is relatively high compared to the other setups for the same treatments.

**Figure 7:**
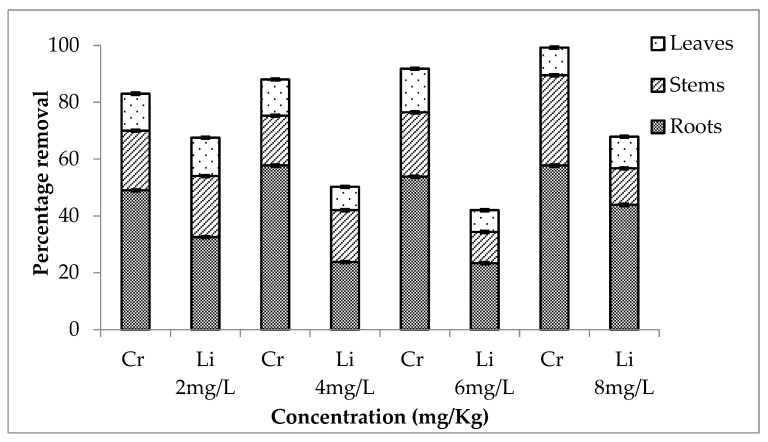
Mean (± SE) percentage removal of Cr + Li by roots, stems and leaves of *Eichhornia crassipes* during first setup where *p* ≤ 0.05.

**Figure 8:**
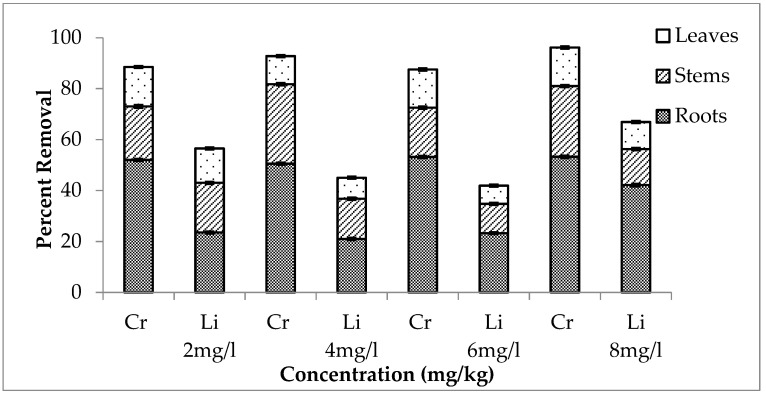
Mean (± SE) percentage removal of Cr + Li by roots, stems and leaves of *Eichhornia crassipes* during second setup where *p* ≤ 0.05.

**Figure 9:**
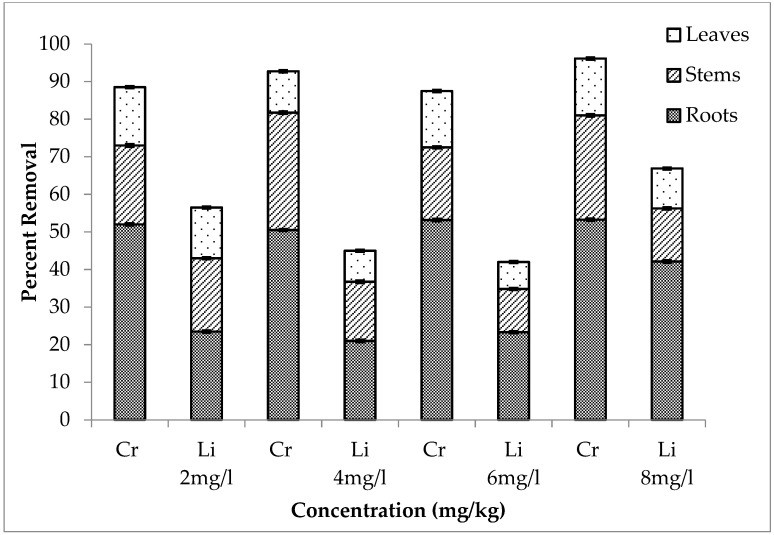
Mean (± SE) percentage removal of Cr + Li by roots, stems and leaves of *Eichhornia crassipes* during third setup where *p* ≤ 0.05.

A combination of chromium and lithium was used to evaluate which element was removed more effectively. It is shown in Figure 7, Figure 8 and Figure 9 that during the first setup, out of total chromium present in the medium, there was 84, 89, 92 and 99% removal for 2, 4, 6 and 8 mg/L, respectively. The percentage removal of lithium during this setup was 69, 40, 42 and 68% (for 2, 4, 6 and 8 mg/L, respectively). Similarly, second and third setup also showed a high removal rate of chromium by *E. crassipes*. The percentage removal of chromium during second setup was 89, 98, 87 and 85% and during third setup, it was 88, 93, 87 and 95% for 2, 4, 6 and 8 mg/L respectively. These results showed that *E. crassipes* is highly efficient for the removal of chromium as compared to lithium.

The concentration of chromium and lithium that was removed by *E. crassipes* was analyzed statistically. One-way ANOVA was applied to the data by the use of SPSS, provided that *p* ≤ 0.05 was the significance value. ANOVA was used to compare the various factors of data. All the results showed that the significance value is less than 0.05, so its means that *E. crassipes* is effective for the removal of both chromium and lithium from the contaminated water at low concentrations, as used in all the present experimental groups. These concentrations are effectively removed by all the parts of the plant (roots, stems and leaves).

### 3.2. Translocation Factor of Cr and Li

Translocation factors of Cr, Li and Cr + Li in *Eichhornia crassipes* were calculated for their different concentrations during the three setups. The TF of Cr and Li are as follows:

Translocation factors of Cr in *Eichhornia crassipes* were calculated for their different concentrations. The TF for Cr during the 3 setups are shown in the figure.

For the first setup, the values of TF were 0.79, 1.16, 0.81 and 0.81 for 2, 4, 6 and 8 mg/L of Cr respectively. These values showed that for 4 mg/L concentration, the Cr is translocated to the aerial parts of the plant, while for other concentrations, Cr remained in the roots of *E. crassipes*. TF is effective for all the concentrations having *p* ≤ 0.5 except for 4 mg/L having a *p*-value greater than 0.05 when compared with the mean of control.

For second setup, the values of TF are 0.47, 0.87, 0.82 and 0.81 for 2, 4, 6 and 8 mg/L of Cr. For all the concentrations, TF is less than 1, which means that most of the Cr remained in the roots. These values are highly effective because all showed a significant value of *p* less than 0.05. Second setup showed more effective TF of Cr as compared to the first one.

**Figure 10:**
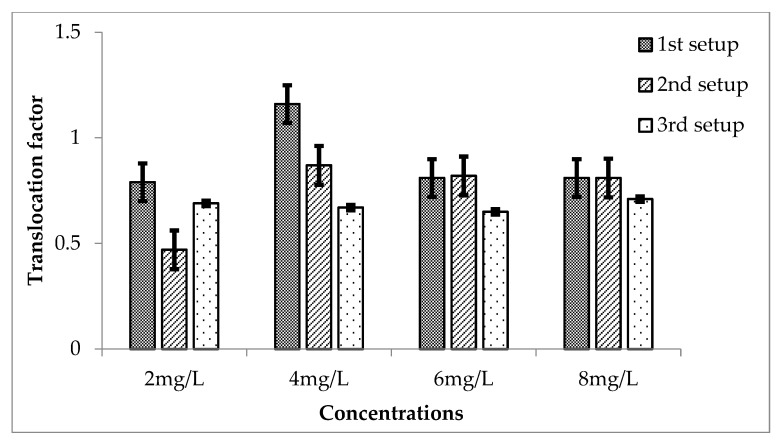
Mean (± SE) translocation factor of Cr in *Eichhornia crassipes* for 3 setups where *p* ≤ 0.05.

Similarly, for second setup, 2, 4, 6 and 8 mg/L showed TF of 0.69, 0.67, 0.65 and 0.71, respectively. TF decreased from 2 to 6 mg/L concentration while increased for 8 mg/L of Cr. These values, when compared with the mean of the control, showed that they are highly significant (*p* ≤ 0.05), as shown in Figure 10.

**Figure 11:**
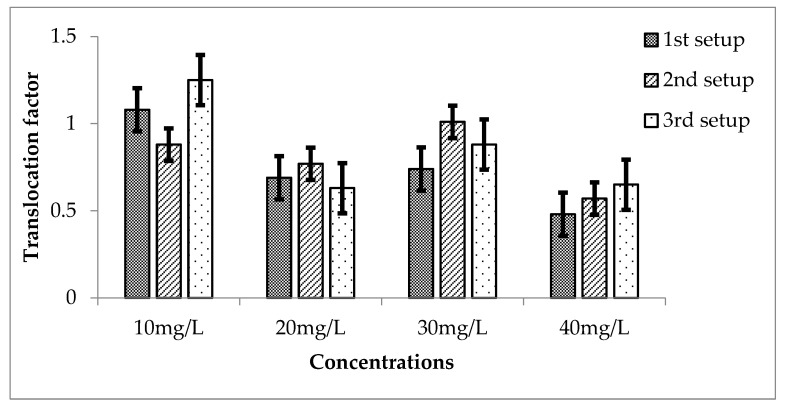
Mean (± SE) translocation factor of Li in *Eichhornia crassipes* for 3 setups where *p* ≤ 0.05.

**Figure 12:**
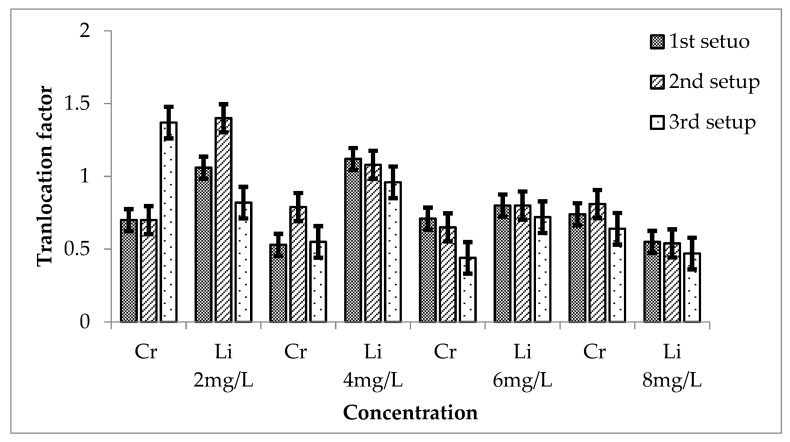
Mean (± SE) translocation factor of Cr + Li in *Eichhornia crassipes* for 3 setups where *p* ≤ 0.05.

The translocation factor of Li in *Eichhornia crassipes* is also calculated for three setups and shown in Figure 11. TF for Li during the 3 setups are shown in Figure 12. For the first setup, TF values were 1.08, 0.69, 0.74 and 0.48 for 10, 20, 30 and 40 mg/L respectively. TF decreased from lower to higher concentrations. Except for 2 mg/L, the values of TF are highly effective, having a *p*-value lesser than 0.05 when compared with the mean of the control, which was 0.

For second setup, 10, 20, 30 and 40 mg/L of Li showed the TF of 0.88, 0.77, 1.01 and 0.57, respectively. This shows that the values decreased from 10 to 40 mg/L except for 30 mg/L, where the values are greater than 1, which showed that Li effectively translocated to the stems and leaves of *E. crassipes*. These are all highly significant because when they are compared with the mean of control its shows that *p* is less than 0.05.

Similarly, for third setup TF was calculated of Li in *E. crassipes*. TF of 1.25, 0.63, 0.88 and 0.65 came out for 10, 20, 30 and 40 mg/L of Li. For 10 mg/L the value of TF is greater than 1.25 which shows the effective translocation to the aerial parts. Other concentrations showed TF less than 1, which is highly significant, having *p* value less than 0.05 when compared with the mean of control, which was 0.

The combination of Cr and Li was also used to evaluate the relative effectiveness of *Eichhornia crassipes* for their removal. TF values are shown in Figure 12 during the 3 setups for Cr + Li.

In the case of first setup, TF values for Cr are 0.70, 0.53, 0.71 and 0.74 for 2, 4, 6 and 8 mg/L, respectively, while for Li, TF were 1.06, 1.12, 0.8 and 0.55 for 2, 4, 6 and 8 mg/L concentration of Li respectively. TF has effective values for all the concentrations of Cr in this case, while for 10 and 20 mg/L of Li, TF is greater than 1, which is not effective, having a significant value greater than 0.05. While for others, TF showed significant values when compared with a mean of control that was 0.

For second setup, TF for 2, 4, 6 and 8 mg/L Cr were 0.70, 0.79, 0.65 and 0.81 respectively. All are lesser than 1 showing a significant value (*p* ≤ 0.05). It shows that Cr remained in the roots and was not much translocated to the aerial parts of the plant. TF of Li was calculated and showed the values of 1.40, 1.08, 0.8 and 0.54 for 2, 4, 6 and 8 mg/L, respectively. The TF of Li was not effective for 2 and 4 mg/L Li showing a value greater than 1. In this case, Li was translocated to the aerial parts of *E. crassipes* and also not effective because *p* ≥ 0.05, when compared with the mean of control, was 0.

Similarly, for third setup, the translocation factor was calculated. The TF for Cr was 1.37, 0.55, 0.44 and 0.64 for 2, 4, 6 and 8 mg/L. the value of TF is greater than 1 for 2 mg/L, which is not much effective because the significant value is greater than 0.05 when compared with the mean of control, which was 0. This shows that at low concentrations, Cr was translocated to the stems and leaves while higher concentrations remained in the roots. TF for Li was 0.82, 0.96, 0.72 and 0.47 for 2, 4, 6 and 8 mg/L of Li. All are significant because when they are compared with the mean of the control, they show *p* ≤ 0.05.

### 3.3. Bioaccumulation Factor of Cr and Li

The bioaccumulation factors of *Eichhornia crassipes* for chromium, lithium and Cr + Li are calculated and described below.

**Figure 13:**
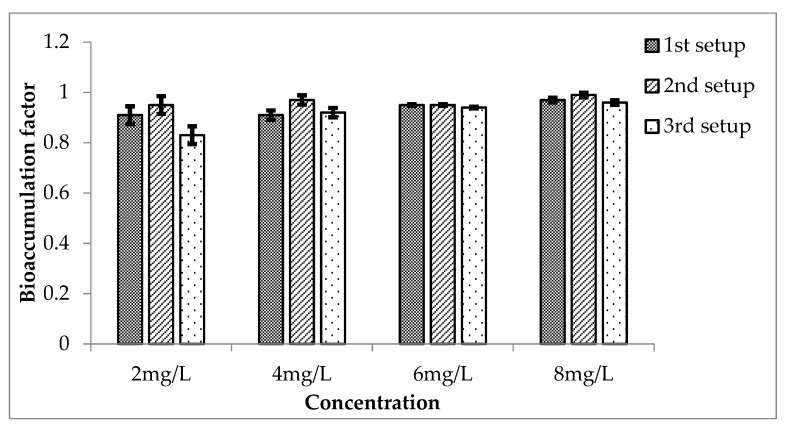
Mean (± SE) bioaccumulation factor of Cr in *Eichhornia crassipes* for 3 setups where *p* ≤ 0.05.

The bioaccumulation factors (BAF) of Cr in *Eichhornia crassipes* for the three setups are shown in Figure 13. These values showed that the values of the factor increased for a high concentration of Cr, and they are highly significant because *p* is less than 0.05 for them when compared with the mean of the control, which was 0. The value increased for 4 mg/L, decreased for 6 mg/L and then increased for 8 mg/L of Cr. These all are highly significant because *p* ≤ 0.05 for all of them when compared with the mean of control, which was 0. The bioaccumulation factor in the case of third setup was also calculated. In this case, the value of the factor is increasing from lower to higher concentrations of Cr. When the BAF was compared with the mean of the control, which was zero, they all showed a significant value of less than 0.05.

**Figure 14:**
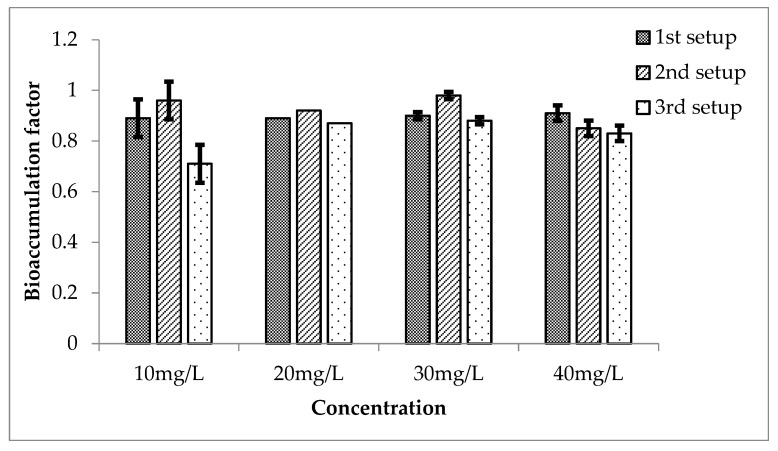
Mean (± SE) bioaccumulation factor of Li in *Eichhornia crassipes* for 3 setups where *p* ≤ 0.05.

The bioaccumulation factors of Li in *Eichhornia crassipes* for the three setups are calculated as shown in Figure 14. During first setup, the value increased for a high concentration of Li, and it is highly significant when analyzed statistically. These results are also highly significant for second and third setups. *E. crassipes* accumulated the metal in it from the water effectively. Statistical analysis of BAF showed that the *p* ≤ 0.05, when compared with the mean of the control, was 0.

**Figure 15:**
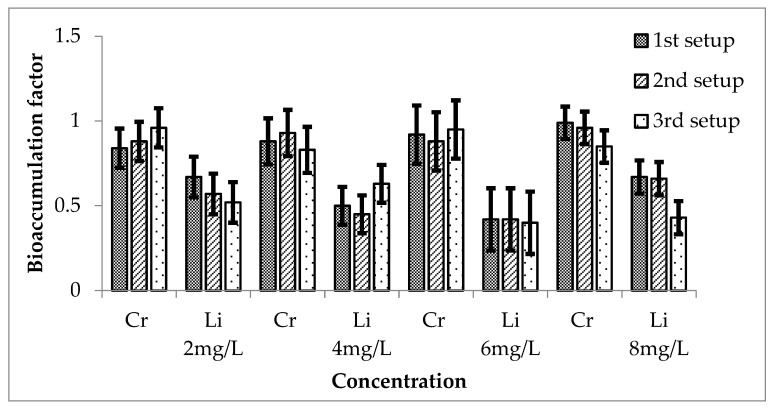
Mean (± SE) bioaccumulation factor of Cr + Li in *Eichhornia crassipes* for 3 setups where *p* ≤ 0.05.

The bioaccumulation factor for a combination of Cr and Li is calculated as shown in Figure 15. The values of BAF for the three setups showed that *E. crassipes* accumulated more concentration of Cr as compared to the Li during all three setups. However, second setup accumulated more concentration of Cr and Li as compared to the other 2 setups. When these values were compared statistically with the mean of the control, they all showed significant results of *p*-values less than 0.05.

## 4. Discussion

The present study evaluated the rate of removal of Cr and Li from contaminated water by the use of *Eichhornia crassipes*. Developing countries are highly dependent on surface water, but it is polluted day by day by heavy metals and other pollutants. Human activities and natural issues are both strains on water resources. The movement of heavy metals and nutrients into the water is also complex [34].

In the present study, 2, 4, 6 and mg/L concentrations of Cr, 10, 20, 30 and 40 mg/L concentrations of Li and 2, 4, 6 and 8 mg/L concentrations of Cr + Li were provided to the triplets of *E. crassipes* in three different experimental setups. Each setup was of 15 days. After 15 days of each setup, plants were harvested, different physiological parameters were measured, and samples were prepared for the analysis of Cr and Li removal by roots, stems and leaves of *E. crassipes*. All the results were analyzed statistically by the use of one-way ANOVA on excel to compare with the mean of the different concentrations. This methodology is in line with [34].

The concentrations of Cr taken for the present study were 2, 4, 6 and 8 mg/L which were also reported before in the literature because these concentrations were effectively removed by *Eichhornia crassipes* [30]. Similarly, low concentrations of Li (10, 20, 30 and 40 mg/L) were also effectively removed by *Eichhornia crassipes* as Li at lower concentrations was effective for the plants without showing negative impacts on their growth [35].

The triplets of control showed an increase in the growth rate, chlorophyll content and other physiological results. However, as the concentration of Cr and Li increased in the medium, negative impacts on the chlorophyll content and other physiological processes were observed. The reason is that *E. crassipes* can grow well in the low concentrations of these elements, but some negative impacts were seen in the high concentrations. It was seen that Li is highly effective for the plant at low concentrations because it did not affect the growth rate and other physiological effects on *E. crassipes*, as it is also reported in the work of [36].

The amounts of Cr and Li removed by the *E. crassipes* were determined by Atomic Absorption Spectrophotometer. The results showed that this aquatic plant could effectively remove the metals accumulated in its body. The roots, leaves and shoots of *E. crassipes* were very significant for the removal of both Cr and Li. A maximum amount of both Cr and Li was absorbed by the roots of *E. crassipes*. While some amounts were also translocated to the stems and leaves, as reported in research works [37].

The roots were not efficient in accumulating the Cr and Li. In the present study, it was seen bioaccumulation factor of the roots was high as compared to the stems and leaves, as it is also reported by [35].

The translocation of Cr and Li to the aerial parts of *E. crassipes* was also calculated from the translocation factor. The TF for all the concentrations of Cr and Li were less than 1. TF value less than one showed that *E. crassipes* could be considered as an excluded family because it keeps Cr and Li away from the roots and stems, as it is also reported by [38].

It is clear from the present results that *Eichhornia crassipes* effectively removed all the concentrations of Cr during all the setups. About 94%, 97%, and 91% Cr were removed after the first, second and third experimental setup, respectively. So this aquatic plant is effective for the removal of heavy metals like Cr. Similar results were reported that 86% Cr was removed by *E. crassipes* during the experiment of 10 days. It not only decreased the concentration of metal in the water but also enhanced the quality of water [39].

Results of the present study demonstrated that in *Eichhornia crassipes* plants, the concentration of Li is lowest in the roots and highest in the leaves, as it is transported from roots to stem to leaves, which are shed when Li concentration becomes high. Moreover, it was observed that when the metal stress increases, then Li is forced to transfer into the stem, where it remains for a short period of time until it is transported further into the leaves.

Some of the impacts observed were the yellowing and shedding of toxic leaves as well as crown burning of plants as a defence mechanism to get rid of excess Li and remain growing. The evident yellowing of leaves started when Li was applied during all threes setups. The yellowing and shedding of leaves and crown burning were much more drastic and apparent in the case of Li application. That is why; it is suggested that there is a need to provide higher doses of Li to *Eichhornia crassipes* to determine the impacts of Li in this specie more clearly; and to observe the Li concentration at which this aquatic plant specie is affected adversely. These findings support the study of [30], who reported that Li damages the health of plants.

The present study is novel because no work has been done before on Li impacts on the growth and physiological parameters of *Eicchornia crassipes* in Pakistan. Internationally as well, there are only a few studies on this subject matter. The removal rate of Cr by *Eichhornia* is also determined because Cr is released by many industries and causes a lot of water pollution. So, Cr is causing a lot of health issues in humans [40]. A combination of Cr and Li is used in the present study to evaluate which element is effectively removed by *E. crassipes*. Therefore, this study emphasizes examining this aquatic plant with respect to metal contamination and one of the emerging pollutants (Li).

This study will be useful for plantation decision-makers in case they require data interrelating Li and the *Eichhornia crassipes*. Moreover, it serves as a stimulator for further studies of Li and other metals on additional native aquatic species of Pakistan. Future studies can acquire assistance from the data and findings of the present study. Additionally, considering this study as a standard; new policies can be made in Pakistan to mitigate the levels of Cr and especially Li prior to its release into the environment. In addition, this study also stimulates the formulation of Li remediation strategies in plant species.

## 5. Conclusions

The present study concludes that *Eichhornia crassipes* can effectively remove Cr and Li at low concentrations. Cr did not show any harmful effects on the plant, while Li showed some negative effects. The effects kept on increasing with increasing doses of Li (up to 40 ppm). Control plants were not affected and kept thriving without any disruption; however, other treatments were affected (10 mg/L at the least and 40 mg/L at the most). Three different experimental setups that were organized showed different rates of removal for Cr and Li. Significant concentrations were removed during the first and second setups as compared to the third setup. In the case of Cr and Li, *Eichornia crassipes* removed relatively more concentration of Cr as compared to Li, showing that *E. crassipes* is more effective for Cr removal. Furthermore, *E. crassipes* was sensitive toward Li as compared to the Cr because its growth and physiological characteristics were reduced, in spite of being provided with the same period of time. The present study was a pot experiment; phytoremediation of Cr and Li must also be conducted on the field level. Due to a restricted period of time for the present study, the rate of removal and the impacts of Cr and Li on only one aquatic species could be determined. Moreover, anatomical and morphological features and the use of other aquatic macrophytes for the removal of these pollutants could also not be explored due to limited time. The physico-analysis of *Eichhornia crassipes* was also conducted due to a limited time period. This study indicates that there is a need for further research on *Eichhornia crassipes* with the provision of increased doses of Cr and Li; so that the impacts of Cr and Li on this specie could be observed more clearly; and also to determine the concentration at which *E. crassipes* starts to lose resistance. Moreover, future research should explore the impacts of Li concentration on more species other than the one in this study. In addition, morphological and anatomical properties of species could also be studied along with physiological characteristics.

This study convinces environmental policymakers to make environmental quality standards that determine a safe concentration of Cr and Li used in different products. This will control Cr and Li contamination in soil and water bodies prior to its release into the environment. Furthermore, data provided in the study will be useful for decision-makers, who may need to select species on the basis of the extent of removal of Cr and Li by them. Moreover, the present study induces the formulation of new, efficient, and working Cr and Li remediation strategies by aquatic plants. This technology could be used for the cleanup of the environment because it is eco-friendly and cost-effective.

## Data Availability

Data is available in the manuscript.
